# Supplementary material for: Co-Occurrence Analysis of Citrus Root Bacterial Microbiota under Citrus Greening Disease
Source: Plants (Basel). 2023 Dec 26;13(1):80. doi: 10.3390/plants13010080 (PMC10781011; doi:10.3390/plants13010080)
Supplement: Supplementary file 1 [file plants-13-00080-s001.zip › Supplementary Figure S1.pdf]

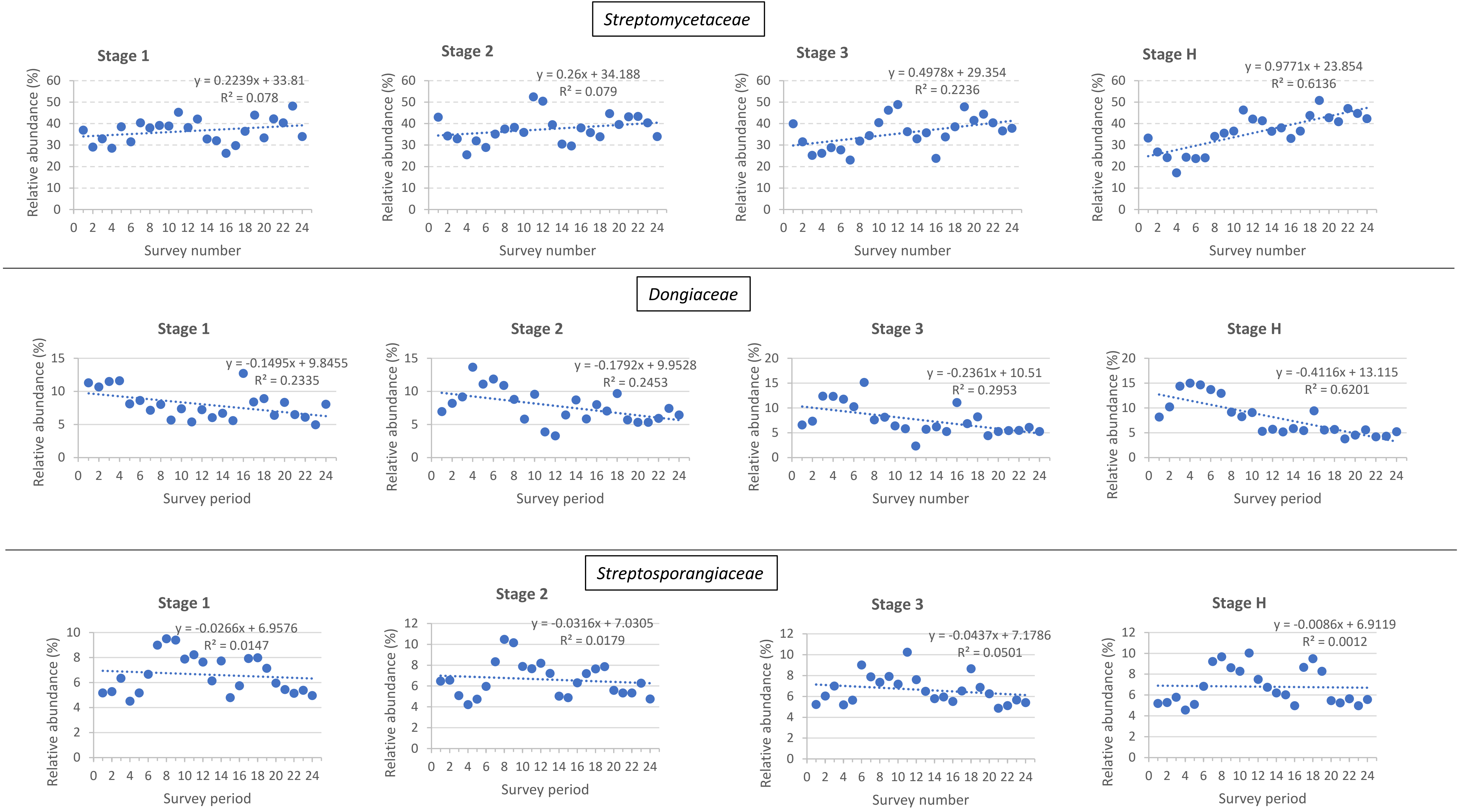
Streptomycetaceae
Stage 1
y = 0.2239x + 33.81
R² = 0.078
Relative abundance (%)
Survey number
Stage 2
y = 0.26x + 34.188
R² = 0.079
Relative abundance (%)
Survey number
Stage 3
y = 0.4978x + 29.354
R² = 0.2236
Relative abundance (%)
Survey number
Stage H
y = 0.9771x + 23.854
R² = 0.6136
Relative abundance (%)
Survey number
Dongiaceae
Stage 1
y = -0.1495x + 9.8455
R² = 0.2335
Relative abundance (%)
Survey period
Stage 2
y = -0.1792x + 9.9528
R² = 0.2453
Relative abundance (%)
Survey period
Stage 3
y = -0.2361x + 10.51
R² = 0.2953
Relative abundance (%)
Survey number
Stage H
y = -0.4116x + 13.115
R² = 0.6201
Relative abundance (%)
Survey period
Streptosporangiaceae
Stage 1
y = -0.0266x + 6.9576
R² = 0.0147
Relative abundance (%)
Survey period
Stage 2
y = -0.0316x + 7.0305
R² = 0.0179
Relative abundance (%)
Survey period
Stage 3
y = -0.0437x + 7.1786
R² = 0.0501
Relative abundance (%)
Survey number
Stage H
y = -0.0086x + 6.9119
R² = 0.0012
Relative abundance (%)
Survey period

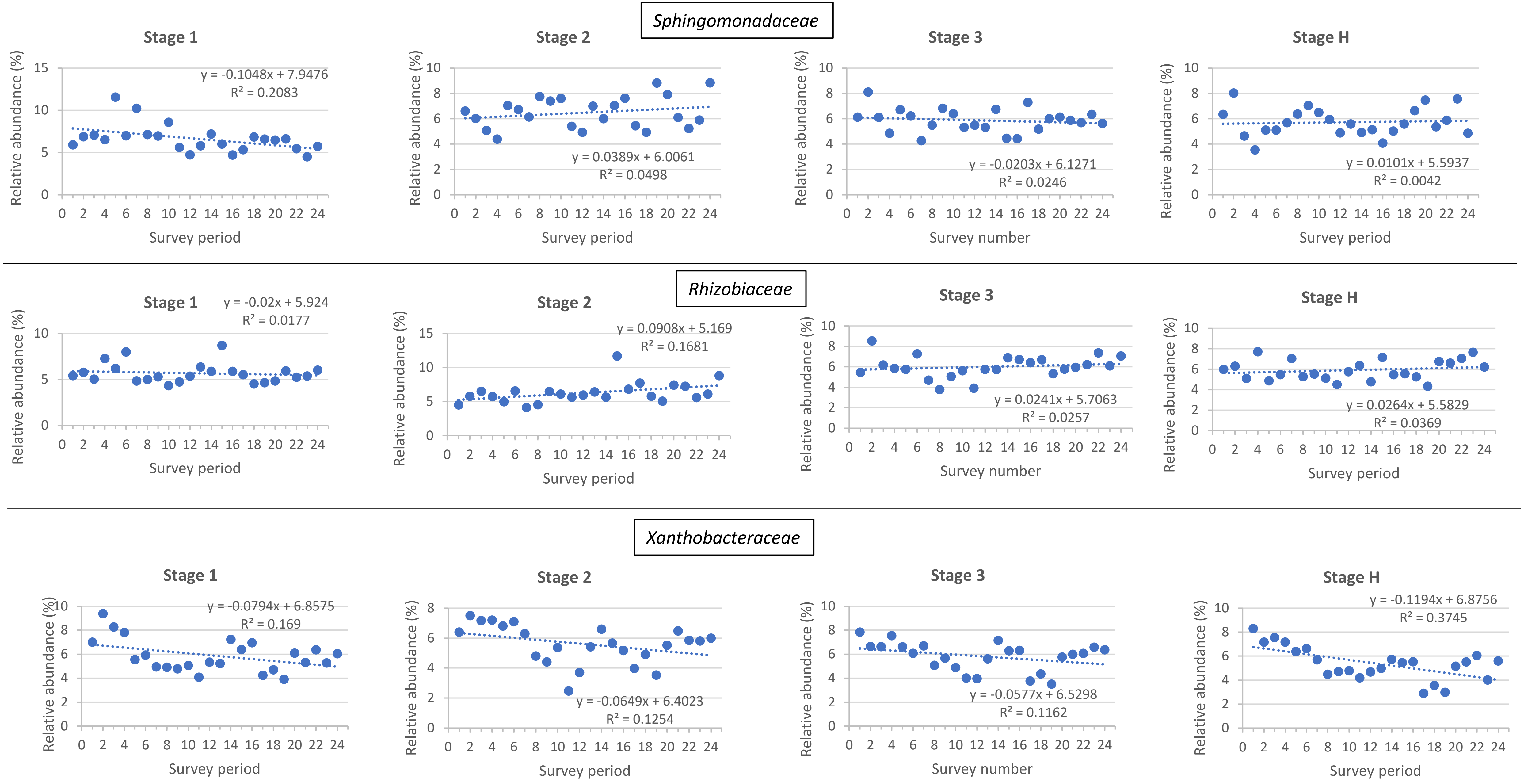

Sphingomonadaceae
Stage 1
y = -0.1048x + 7.9476
R² = 0.2083
Relative abundance (%)
Survey period
Stage 2
y = 0.0389x + 6.0061
R² = 0.0498
Relative abundance (%)
Survey period
Stage 3
y = -0.0203x + 6.1271
R² = 0.0246
Relative abundance (%)
Survey number
Stage H
y = 0.0101x + 5.5937
R² = 0.0042
Relative abundance (%)
Survey period
Rhizobiaceae
Stage 1
y = -0.02x + 5.924
R² = 0.0177
Relative abundance (%)
Survey period
Stage 2
y = 0.0908x + 5.169
R² = 0.1681
Relative abundance (%)
Survey period
Stage 3
y = 0.0241x + 5.7063
R² = 0.0257
Relative abundance (%)
Survey number
Stage H
y = 0.0264x + 5.5829
R² = 0.0369
Relative abundance (%)
Survey period
Xanthobacteraceae
Stage 1
y = -0.0794x + 6.8575
R² = 0.169
Relative abundance (%)
Survey period
Stage 2
y = -0.0649x + 6.4023
R² = 0.1254
Relative abundance (%)
Survey period
Stage 3
y = -0.0577x + 6.5298
R² = 0.1162
Relative abundance (%)
Survey number
Stage H
y = -0.1194x + 6.8756
R² = 0.3745
Relative abundance (%)
Survey period

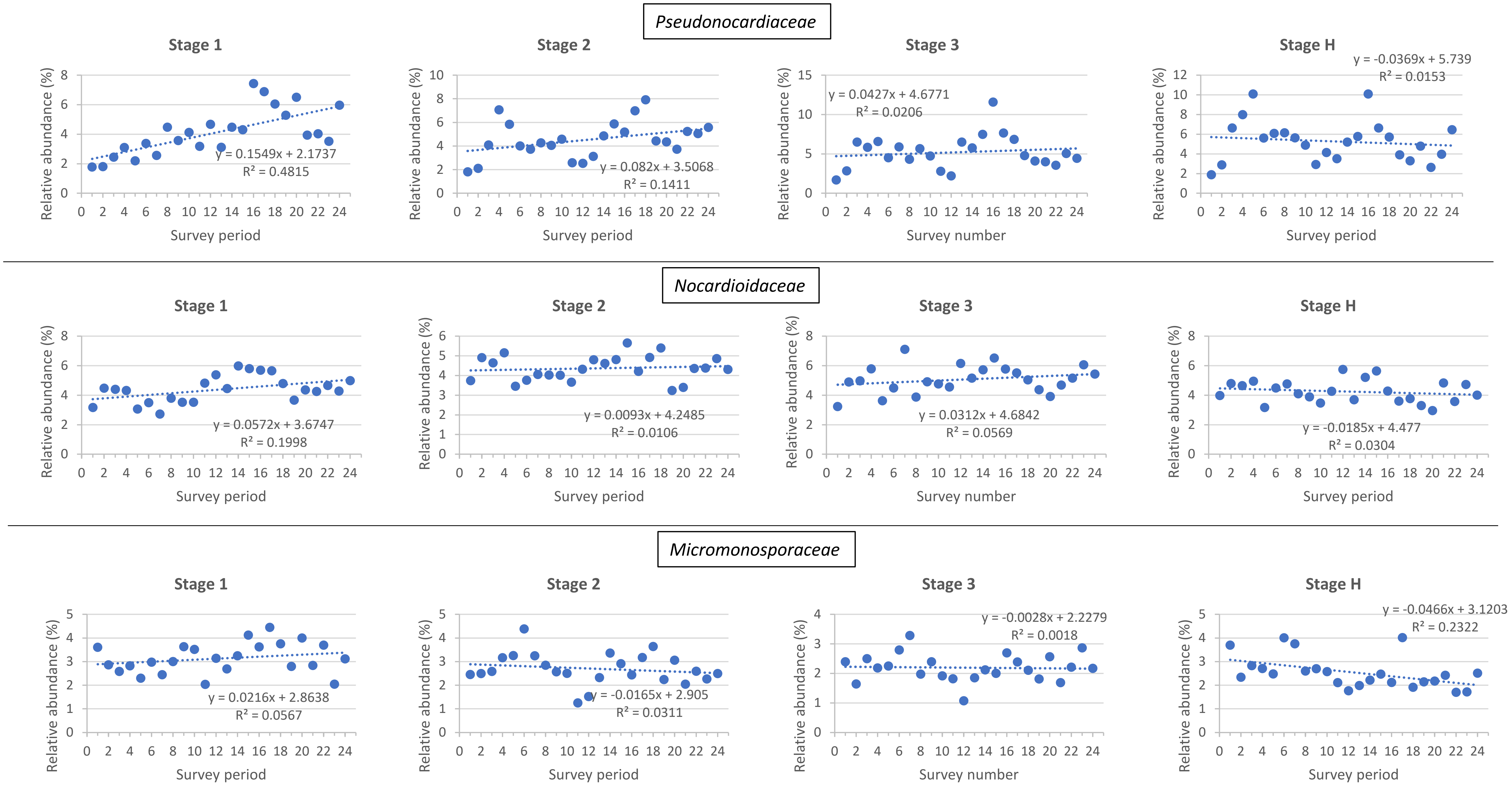
Pseudonocardiaceae
Stage 1
y = 0.1549x + 2.1737
R² = 0.4815
Stage 2
y = 0.082x + 3.5068
R² = 0.1411
Stage 3
y = 0.0427x + 4.6771
R² = 0.0206
Stage H
y = -0.0369x + 5.739
R² = 0.0153
Nocardioidaceae
Stage 1
y = 0.0572x + 3.6747
R² = 0.1998
Stage 2
y = 0.0093x + 4.2485
R² = 0.0106
Stage 3
y = 0.0312x + 4.6842
R² = 0.0569
Stage H
y = -0.0185x + 4.477
R² = 0.0304
Micromonosporaceae
Stage 1
y = 0.0216x + 2.8638
R² = 0.0567
Stage 2
y = -0.0165x + 2.905
R² = 0.0311
Stage 3
y = -0.0028x + 2.2279
R² = 0.0018
Stage H
y = -0.0466x + 3.1203
R² = 0.2322
Relative abundance (%)
Survey period
Survey number

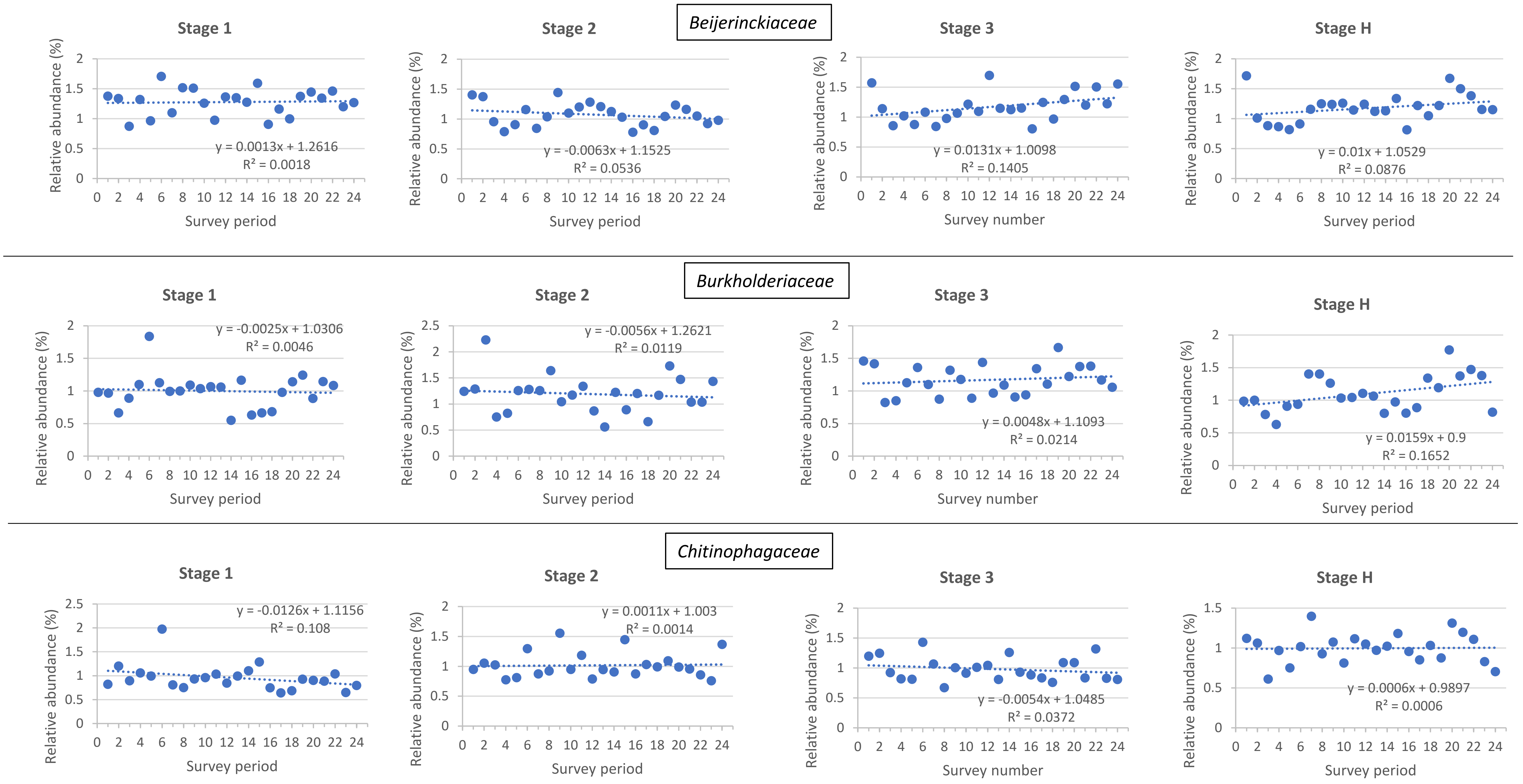

Figure S1. The changes in the relative abundance of twelve bacterial families with ≥ ~1% relative abundance over 24 consecutive monthly survey periods. The linear regression equation and R square value were indicated in the figure.
