# Supplementary material for: Co-Occurrence Analysis of Citrus Root Bacterial Microbiota under Citrus Greening Disease
Source: Plants (Basel). 2023 Dec 26;13(1):80. doi: 10.3390/plants13010080 (PMC10781011; doi:10.3390/plants13010080)
Supplement: Supplementary file 1 [file plants-13-00080-s001.zip › Supplementary Figure S2.pdf]

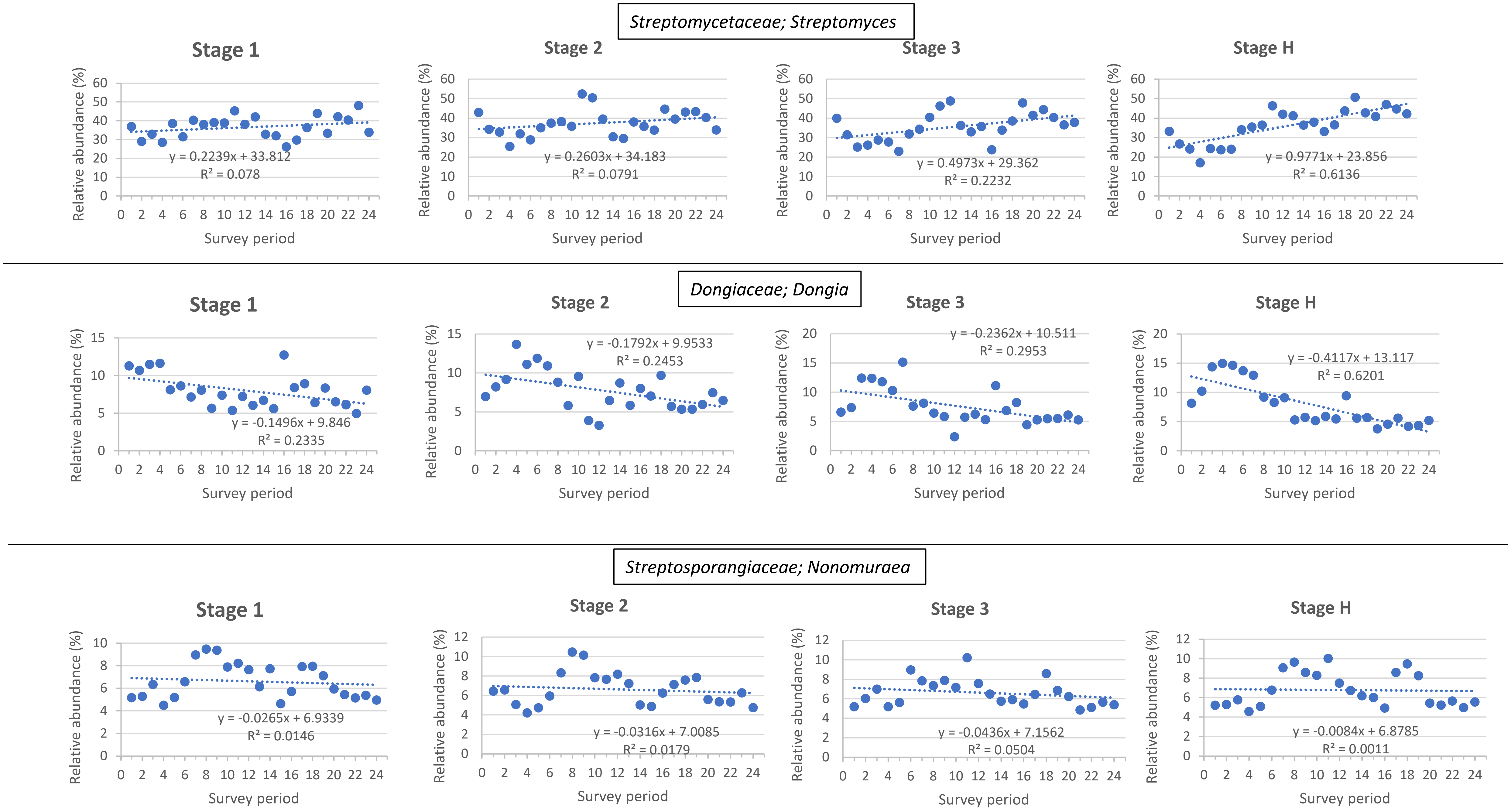

Streptomycetaceae; Streptomyces
Stage 1
y = 0.2239x + 33.812
R² = 0.078
Stage 2
y = 0.2603x + 34.183
R² = 0.0791
Stage 3
y = 0.4973x + 29.362
R² = 0.2232
Stage H
y = 0.9771x + 23.856
R² = 0.6136
Dongiaceae; Dongia
Stage 1
y = -0.1496x + 9.846
R² = 0.2335
Stage 2
y = -0.1792x + 9.9533
R² = 0.2453
Stage 3
y = -0.2362x + 10.511
R² = 0.2953
Stage H
y = -0.4117x + 13.117
R² = 0.6201
Streptosporangiaceae; Nonomuraea
Stage 1
y = -0.0265x + 6.9339
R² = 0.0146
Stage 2
y = -0.0316x + 7.0085
R² = 0.0179
Stage 3
y = -0.0436x + 7.1562
R² = 0.0504
Stage H
y = -0.0084x + 6.8785
R² = 0.0011
Relative abundance (%)
Survey period

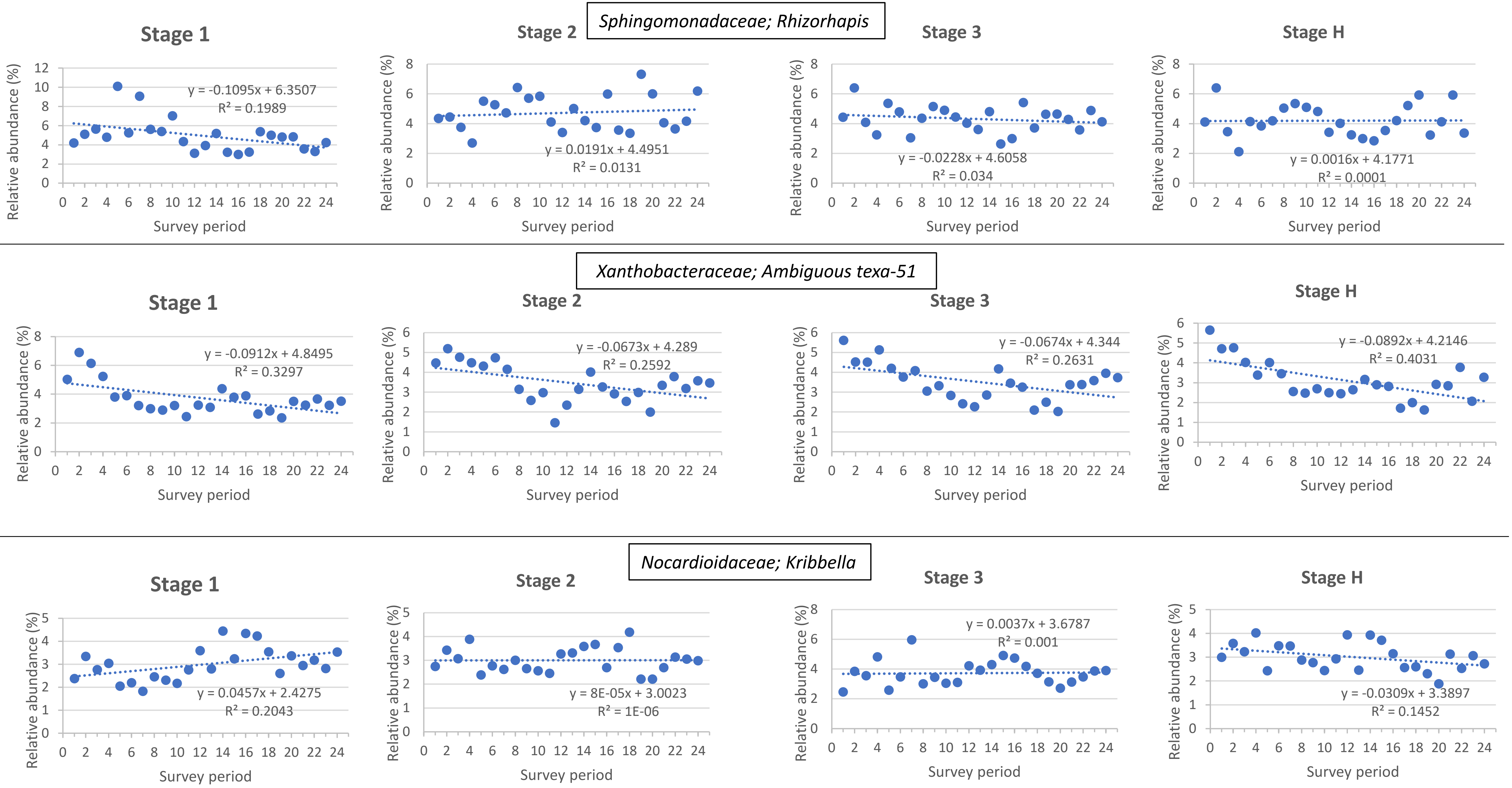
Sphingomonadaceae; Rhizorhapis
Stage 1
y = -0.1095x + 6.3507
R² = 0.1989
Stage 2
y = 0.0191x + 4.4951
R² = 0.0131
Stage 3
y = -0.0228x + 4.6058
R² = 0.034
Stage H
y = 0.0016x + 4.1771
R² = 0.0001
Xanthobacteraceae; Ambiguous texa-51
Stage 1
y = -0.0912x + 4.8495
R² = 0.3297
Stage 2
y = -0.0673x + 4.289
R² = 0.2592
Stage 3
y = -0.0674x + 4.344
R² = 0.2631
Stage H
y = -0.0892x + 4.2146
R² = 0.4031
Nocardioidaceae; Kribbella
Stage 1
y = 0.0457x + 2.4275
R² = 0.2043
Stage 2
y = 8E-05x + 3.0023
R² = 1E-06
Stage 3
y = 0.0037x + 3.6787
R² = 0.001
Stage H
y = -0.0309x + 3.3897
R² = 0.1452
Relative abundance (%)
Survey period

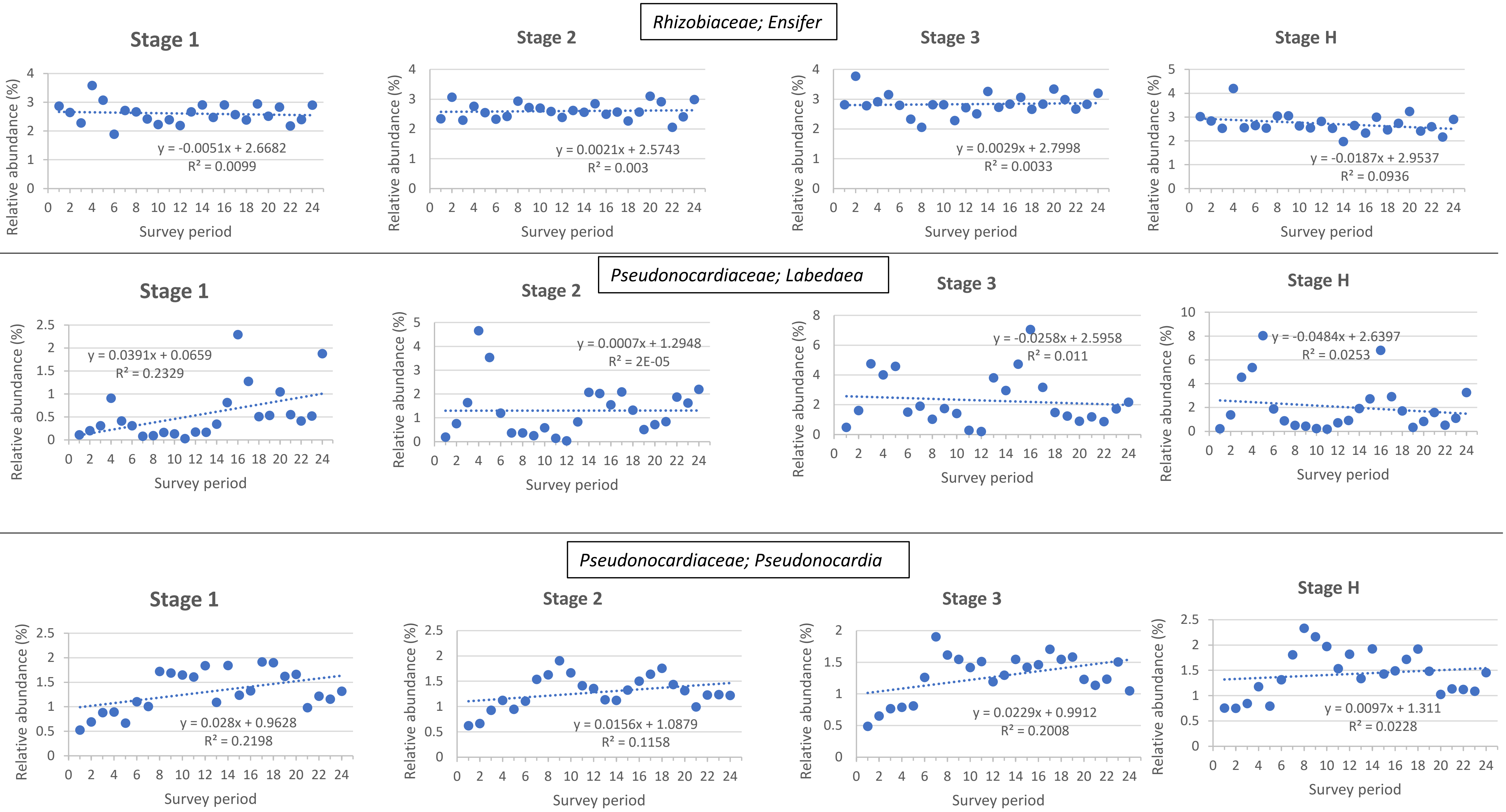
Rhizobiaceae; Ensifer
Stage 1
y = -0.0051x + 2.6682
R² = 0.0099
Stage 2
y = 0.0021x + 2.5743
R² = 0.003
Stage 3
y = 0.0029x + 2.7998
R² = 0.0033
Stage H
y = -0.0187x + 2.9537
R² = 0.0936
Pseudonocardiaceae; Labedaea
Stage 1
y = 0.0391x + 0.0659
R² = 0.2329
Stage 2
y = 0.0007x + 1.2948
R² = 2E-05
Stage 3
y = -0.0258x + 2.5958
R² = 0.011
Stage H
y = -0.0484x + 2.6397
R² = 0.0253
Pseudonocardiaceae; Pseudonocardia
Stage 1
y = 0.028x + 0.9628
R² = 0.2198
Stage 2
y = 0.0156x + 1.0879
R² = 0.1158
Stage 3
y = 0.0229x + 0.9912
R² = 0.2008
Stage H
y = 0.0097x + 1.311
R² = 0.0228
Relative abundance (%)
Survey period

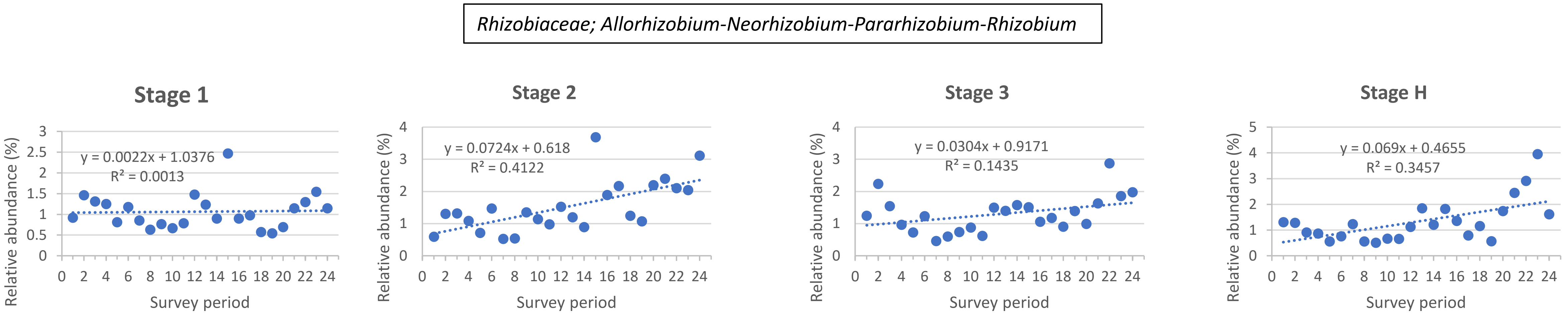

Figure S2. The changes in the relative abundance of top ten most abundant bacterial genera over 24 consecutive monthly survey periods. The linear regression equation and R square value were indicated in the figure.
