# Supplementary material for: Co-Occurrence Analysis of Citrus Root Bacterial Microbiota under Citrus Greening Disease
Source: Plants (Basel). 2023 Dec 26;13(1):80. doi: 10.3390/plants13010080 (PMC10781011; doi:10.3390/plants13010080)
Supplement: Supplementary file 1 [file plants-13-00080-s001.zip › Supplementary Figure S3.pdf]

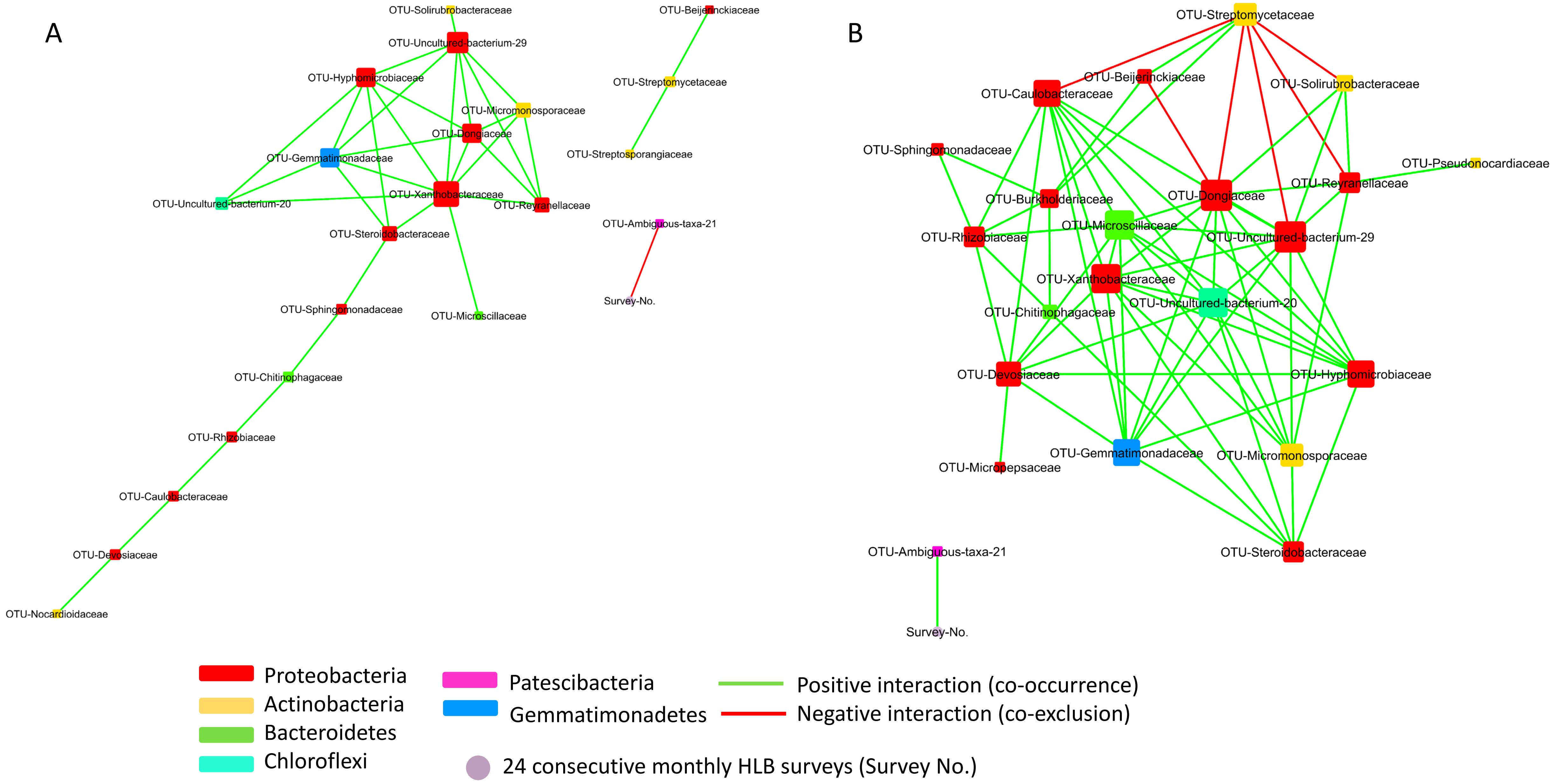

Figure S3. Co-occurrence network analysis of citrus root bacterial microbiota with > 0.3% relative abundance at the family level in the trees of Stage II (A) and Stage III (B). Node size and color indicated the degree (number of connections) and bacterial family, respectively. The positive and negative interactions were indicated in green and red lines.
