# Supplementary material for: Co-Occurrence Analysis of Citrus Root Bacterial Microbiota under Citrus Greening Disease
Source: Plants (Basel). 2023 Dec 26;13(1):80. doi: 10.3390/plants13010080 (PMC10781011; doi:10.3390/plants13010080)
Supplement: Supplementary file 1 [file plants-13-00080-s001.zip › Supplementary Figure S4.pdf]

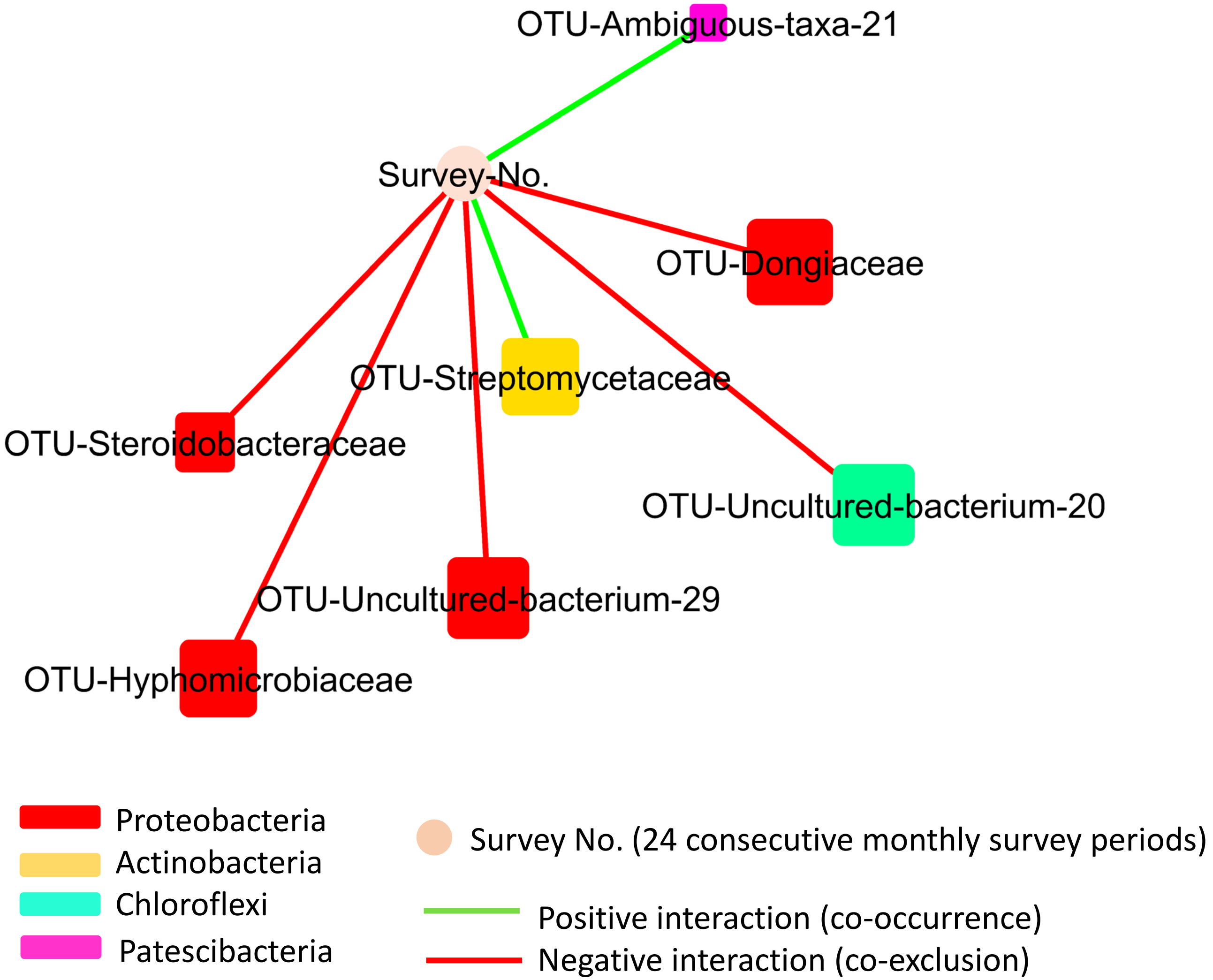

Figure S4. Stage H co-occurrence network showing the correlation of seven bacterial families with 24 consecutive monthly surveys.
